# Evaluation of atrial fibrosis in atrial fibrillation patients with three different methods

**DOI:** 10.3906/sag-2103-194

**Published:** 2021-09-20

**Authors:** Cem ÇÖTELİ, Tuncay HAZIROLAN, Kudret AYTEMİR, Ahmet Gürkan ERDEMİR, Emine Nilay BAKIR, Uğur CANPOLAT, Hikmet YORGUN, Ahmet Hakan ATEŞ, Ergun Barış KAYA, Zeliha Günnur DİKMEN, Necla ÖZER

**Affiliations:** 1 Department of Cardiology, Ankara City Hospital, Ankara, Turkey; 2 Department of Radiology, Faculty of Medicine, Hacettepe University, Ankara, Turkey; 3 Department of Cardiology, Faculty of Medicine, Hacettepe University, Ankara, Turkey; 4 Department of Biochemistry, Faculty of Medicine, Hacettepe University, Ankara, Turkey; 5 Texas Cardiac Arrhythmia Institute, St. David’s Medical Center, Austin, TX, USA; 6 Department of Cardiology, Faculty of Health Medicine and Life Sciences, Maastricht University, Maastricht, Netherland

**Keywords:** Atrial fibrillation, atrial fibrosis, cardiac MRI T1 relaxation duration, FGF-21, FGF-23, atrial strain imaging

## Abstract

**Background/aim:**

The presence of atrial fibrosis has already been known as a risk factor for atrial fibrillation (AF) development. We aimed to evaluate atrial fibrosis with previously defined three different methods, which were cardiac magnetic resonance imaging (C-MRI), echocardiographic strain imaging, and biomarkers and show the relationship between these methods in patients with AF scheduled for cryoballoon ablation.

**Materials and methods:**

A total of 30 patients were enrolled. Atrial T1 relaxation durations were measured using C-MRI before the procedure of atrial fibrillation catheter ablation. Fibroblast growth factor-21 (FGF-21) and fibroblast growth factor-23 (FGF-23) levels were measured at serum derived from the femoral artery (Peripheral FGF 21 and 23) and left atrium blood samples (Central FGF 21 and 23) before catheter ablation. Preprocedural transthoracic echocardiography was performed. The median follow-up duration for atrial tachyarrhythmia (ATa) recurrence was 13 (12–18 months) months.

**Results:**

The mean ages of the study group were 55.23 ± 12.37 years, and there were 17 (56.7%) female patients in study population. There were negative correlations between post contrast T1 relaxation durations of both posterior and posterosuperior atrium, and central FGF-23 (r: − 0.561; p = 0.003; r: −0.624; p = 0.001; Posterior T1 vs. central FGF-23 levels and Posterosuperior T1 vs central FGF-23 levels, respectively). The positive correlations were observed between postcontrast posterior T1 relaxation durations and left ventricle ejection fraction (r:0.671; p = 0.001); left atrial emptying fraction (r:0.482; p = 0.013); peak atrial longitudinal strain (r:0.605; p = 0.001), and peak atrial contraction strain (r:0.604; p = 0.001). Also negative correlation was observed between postcontrast posterior T1 relaxation durations, and left atrial volume index (r: − 0.467; p = 0.016).

**Conclusion:**

Our studies showed that there are correlations between T1 mapping of atrial tissue, biomarkers, and atrial mechanics evaluated with transthoracic echocardiography. Our findings are significant as they demonstrate the compatibility of these techniques for the atrial fibrosis evaluation.

## 1. Introduction

Catheter ablation for atrial fibrillation (AF) is a well-known effective and safe therapeutic option, particularly in paroxysmal AF. The standard therapy primarily targets the isolation of pulmonary veins (PVI) [1, 2]. However, arrhythmia recurs in ~30 to 40% of patients after an index procedure[3]. Thus, several measures, including imaging techniques and biomarkers predicting the recurrence after catheter ablation have been developed.

The presence of atrial fibrosis has already been known as a risk factor for AF development [4]. Atrial fibrosis is one of the good predictors of catheter ablation success [5]. Various invasive or noninvasive techniques can demonstrate the presence and severity of atrial fibrosis. Delayed enhancement magnetic resonance imaging (DE-MRI) is a popular method to show the presence and severity of atrial fibrosis in recent AF ablation studies [6, 7]. However, there is no standard software for the evaluation of left atrium with high reproducibility and global availability. T1 mapping is a recently found method to assess the physiological and pathophysiological alterations in native T1 and extracellular volume [8]. Cardiac magnetic resonance imaging (C-MRI) T1 imaging is used for the assessment of myocardial fibrosis. This method can predict the success of AF ablation [9–11]. Furthermore, there are some novel biomarkers related to atrial fibrosis. It has been shown that changes in the levels of serum FGF (Fibroblast Growth Factor)-21 and FGF-23 are significant to foretell the occurrence of AF [12, 13].

In this study, we aimed to assess the correlations between atrial fibrosis indicators including C-MRI T1 mapping, echocardiographic parameters, serum FGF-21/FGF-23 levels in patients who had been scheduled for AF ablation using second-generation cryoballoon (CB).

## 2. Materials and methods

### 2.1. Study population

In this prospective observational study, we enrolled 30 patients with paroxysmal AF who underwent CB catheter ablation between May 2017 and October 2017 in Hacettepe University, Department of Cardiology, Electrophysiology Unit. All patients had recurrent symptomatic paroxysmal AF despite the use of at least one antiarrhythmic drug, and they were scheduled for catheter ablation for AF. Patients with uncontrolled thyroid function, nonparoxysmal AF, advanced heart failure, systemic infectious or inflammatory disease, chronic renal or liver failure, dilated cardiomyopathy, severe valvular disease, previous history of AF ablation, left atrial thrombus, presence of a permanent pacemaker or implantable cardioverter-defibrillator were excluded. Paroxysmal AF was defined as an episode of AF that terminates spontaneously or with intervention within seven days [2].

**Figure 1:**
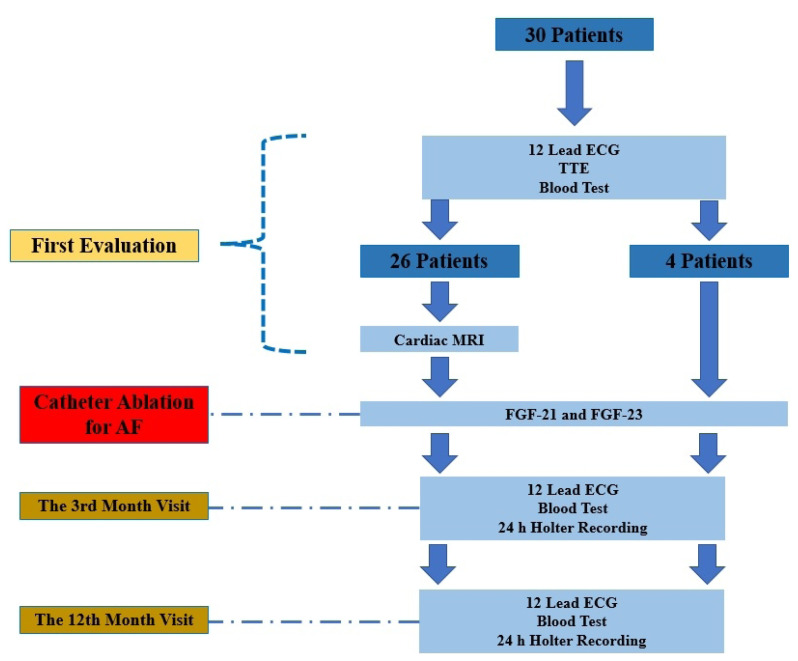
The flowchart of the study.

Detailed medical history was taken from each patient and the demographic, clinical, and laboratory data of the participants were recorded. All patients were evaluated comprehensively with 12-derived electrocardiography, transthoracic echocardiography (TTE), and routine laboratory tests on index admission. Additionally, C-MRI was performed one week before the catheter ablation procedure. Blood samples for FGF-21 & FGF-23 were taken from the femoral artery and left atrium just before catheter ablation. The flowchart of the study was represented in Figure 1.

Informed consent was taken from each patient before the procedure. The study was in compliance with the principles outlined in the Declaration of Helsinki. The institutional ethics committee approved this study.

### 2.2. Transthoracic echocardiography

Transthoracic echocardiography was performed with the GE Vivid E9 (GE Healthcare, Horten, Norway) device one week before the CB catheter ablation procedure. The cardiologist who performed TTE was blinded to the clinical data and experienced in cardiac imaging. Intra-observer reliability was reported in Supplementary Table 1. In compliance with the recommendations of the European Association of Cardiovascular Imaging (EACVI), two-dimensional imaging examination and chamber quantification were recorded [14]. Conventional left ventricular systolic function parameters, including left ventricle end-diastolic diameter (LVEDD) and left ventricle ejection fraction (LVEF), were calculated. We used the Biplane Simpson Method for the LVEF calculation [14].

All views were recorded in five consecutive beats at the end of expiration, and the frame rate was set to be more than 60 frames per second. The left ventricle myocardium was selected as the region of interest (ROI) for the left ventricle global longitudinal strain (GLS) calculation. The width of the ROI was set between 2 to 10 mm. The entire endocardium was traced manually in all patients. We used GE EchoPAC Clinical Workstation Software for drawing the graphs and bullseye images, and calculation of left ventricle global longitudinal peak strain values. Endocardial strain values were used for statistical analysis. Strain values were expressed as negative percentage values.

**Figure 2:**
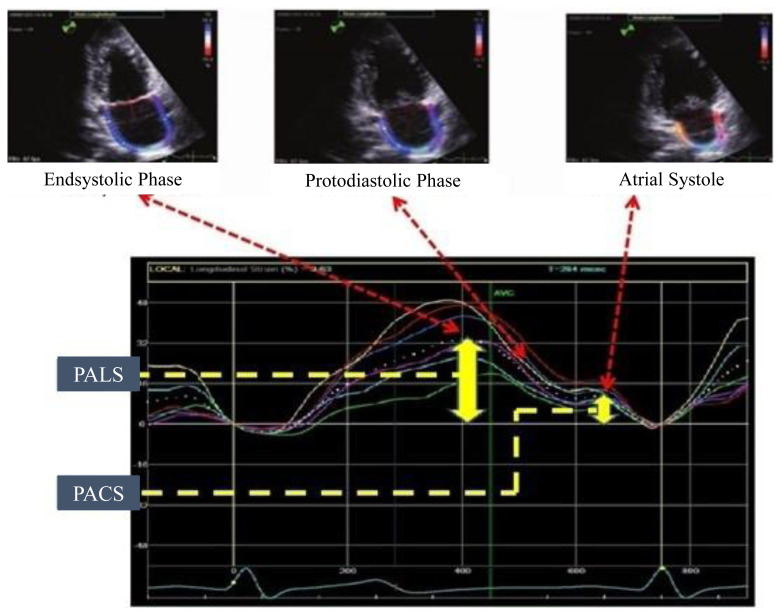
Left atrial strain evaluation. Strain curves of different left atrial walls are represented in different colors. The global strain curve of the left atrium is represented as a white dashed curve. *PALS:* Peak atrial longitudinal strain, the peak value of positive wave at end-systolic phase. *PACS:* Peak atrial contraction strain, the peak value of positive wave at atrial systolic phase.

Left atrial tissue was selected as the ROI to calculate left atrial longitudinal strain values. The width of ROI was set between 2 to 5 mm. Entire left atrial tissue was traced manually in all patients. The graphs of atrial longitudinal strain values were drawn for apical four-chamber and apical two-chamber. The same software was used for the calculation of mean values and drawing the diagrams. Left atrial strain values were calculated as PALS, PACS, and the portion of PACS to PALS (contraction strain index-CSI). PALS is the first positive peak value at the RR interval and positive peak value at the end of the reservoir phase. PACS is the second positive peak following the landing of the first peak value and is right before the beginning of atrial contraction (Figure 2).

We used the biplane area-length method for the calculation of the maximum left atrial volume (VmaxA), the minimum left atrial volume (VminA), and left atrial precontraction volume (VpreA). Moreover, we calculated the left atrial volume index (LAVI), left atrial emptying fraction (LAEmF), left atrial active emptying fraction (LAAEmF), and left atrial passive emptying fraction (LAPEmF).

### 2.3. Cardiac magnetic resonance imaging

In 26 patients, C-MRI was performed one week before the catheter ablation procedure using Philips Ingenia CX 1.5 Tesla MRI device (Philips Ingenia CX, Amsterdam, Netherlands). In four patients, C-MRI could not be performed, because of tachycardia episodes during the MRI procedure and agoraphobia. All patients were in sinus rhythm during the test. Pre-contrast and post-contrast single-shot echo sequence views were captured for sagittal-oblique and transverse planes. MOLLI (Modified Lock-Locker) based software, which is available in the workstation of the MRI device, was used for T1 quantification.

Because of the thin nature of the atrial myocardium, we decided to use relatively thicker left atrial tissue - posterior and posterosuperior left atrial myocardium- for atrial T1 quantification [8, 9]. An experienced radiologist (T.H.), who was blinded to the clinical data, calculated the T1 values in all patients. Intra-observer reliability coefficients were measured 0.953 and 0.957 for postcontrast T1 relaxation durations of the posterior atrium and posterosuperior atrium, respectively (Supplementary Table 1).

**Figure 3:**
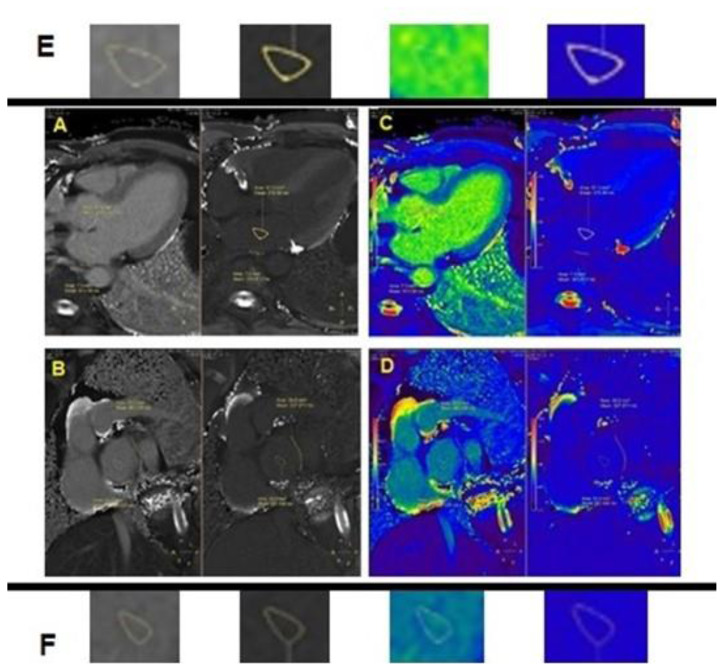
**A:** Gray scale of posterior atrium. **B:** Gray scale of posterosuperior atrium. **C:** Rainbow-colored of posterior atrium. **D:** Rainbow-colored of posterosuperior atrium. **E:** Samples for T1 quantification of posterior atrium, selected in same shape and width. **F:** Samples for T1 quantification of posterosuperior atrium, selected in same shape and width.

The areas were selected as in the same width and shape to protect objectivity. An example of T1 mapping was shown in Figure 3. Gray scale images in Figures 3A and 3B, rainbow-colored images in Figures 3C and 3D, samples for T1 quantifications in Figures 3E and 3F were presented.

### 2.4. Catheter ablation of AF

The CB catheter ablation of AF was performed according to the similar steps applied by Canpolat et al.’s study [15]. Briefly, all procedures were performed under conscious sedation. Transseptal puncture was performed with modified Brockenbrough technique (BRK-1, St. Jude Medical, St. Paul, MN, USA) and with fluoroscopy guidance. After a successful transseptal puncture, unfractionated heparin boluses were administered to maintain the activated clotting time of 300–350 s. All procedures have been performed by using the 28-mm second-generation CB catheter (Arctic Front AdvanceTM, Medtronic Inc., Minneapolis, MN, USA). Pulmonary vein conduction was evaluated by the circular mapping catheter (15-mm Achieve TM; Medtronic Inc., Minneapolis, MN, USA) during the procedure. Successful pulmonary vein isolation was defined as the elimination or dissociation of all the visible pulmonary vein potentials recorded by the circular mapping catheter.

### 2.5. Blood sampling and calculation of FGF-21 and FGF-23

In all patients, peripheral arterial blood was drawn from the femoral artery and central blood samples were taken from the left atrium. FGF-21 and FGF-23 are hormones and secreted from different tissues. To evaluate the systemic and atrial measures of these hormones, we decided to take blood from two different locations. We had waited for 30 min for the completion of coagulation after sampling. Then, tubes had been centrifuged at 1000g for 15 min with NUVE NF 1200 R centrifugation device (Nüve, Turkey). Divided blood samples were stored at −70 °C until biochemical study for FGF-21 and FGF-23. We used the Biovendor human-sourced ELISA kit (BioVendor, Czech) for the test of FGF-21 (Intra-assay CV = 2.0%; Inter-assay CV = 3.3%) and Aviscera Bioscience human-sourced kit (Aviscera Bioscience, USA) for the test of FGF-23 (Intra-assay CV = 6–8%; Inter-assay CV = 10%–12%). FGF-21 and FGF-23 levels of central and peripheral samples were calculated with the sandwich ELISA method. All calculation was performed with TECAN Sunrise microplate reader (Tecan Sunrise, Austria).

### 2.6. Statistical analysis

**Figure 4:**
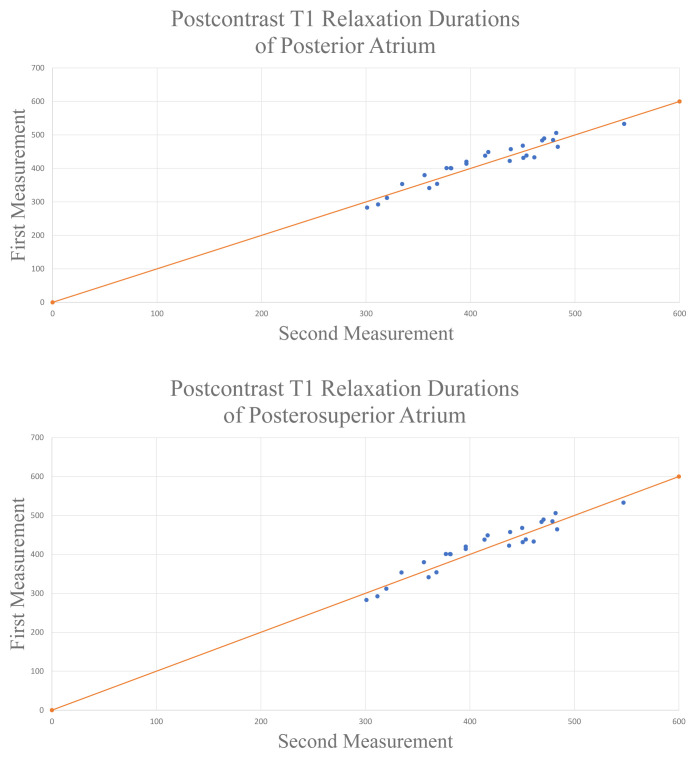
Lin’s correlation graphs for postcontrast T1 relaxation durations of the posterior atrium in **A** and postcontrast T1 relaxation durations of the posterosuperior atrium in **B**.

The NCCS (Number Cruncher Statistical System, 2007, Kaysville, Utah, USA) was used for all statistical analyses. Descriptive and categorical variables were presented as frequencies and percentages. Continuous data with normal distribution were expressed as means ± SD. Quantitative variables without normal distribution were described as median and min-max range. Pearson Correlation test was used for the determination of the variables’ relationship. Paired sample t-test was used for in-group comparison of the variables with a normal distribution. Patients’ echocardiographic evaluations before and 12 months after the CB ablation were compared with paired sample T-test. Intra-observer reliability was assessed with the intraclass correlation coefficient postcontrast T1 relaxation durations and atrial strain values. Additionally, Lin’s concordance correlation coefficients and graphs were examined to assess intra-observer agreements for quantification of postcontrast T1 durations. A 95% level of agreement was reported for both postcontrast T1 relaxation durations and atrial strain value (Figure 4; 4A for posterior atrium and 4B for posterosuperior atrium). At least p < 0.05 is accepted for statistical significance.

## 3. Results

### 3.1. Baseline characteristics

Thirty patients who underwent catheter ablation for atrial fibrillation were included in the study. All patients had paroxysmal atrial fibrillation, and none of them had a history of catheter ablation. The mean ages of the study group were 55.23 ± 12.37 years, and the most common comorbidity, which was present in 19 (63.3%) patients, was hyperlipidemia. The baseline characteristics of the study population were detailed in Table 1.

### 3.2. Outcomes of catheter ablation of AF

During a median of 13 months (12–18 months) follow-up, ATa recurrence was observed in four patients (13.3%). Two of these patients had undergone a re-do procedure.

### 3.3. Atrial fibrosis evaluation in three different methods

All patients were evaluated with echocardiography, and serum FGF-21 and FGF-23 levels were measured in all. C-MRI was performed in 26 of the patients. Results were listed in Table 2.

### 3.4. Correlations between C-MRI T1 relaxation durations and biomarkers

There were negative correlations between post-contrast T1 relaxation durations of both posterior (r: − 0.561; p = 0.003) and posterosuperior atrium (r: − 0.624; p = 0.001), and central FGF-23 level. The results of correlation analysis between atrial T1 relaxation durations and biomarkers, which are FGF-21 and 23, were presented in Table 3.

### 3.5. Correlations between C-MRI T1 relaxation durations and transthoracic echocardiographic findings

The positive correlations were observed between postcontrast posterior T1 relaxation durations and LVEF (r:0.671; p = 0.001); LAAEmpF (r:0.482; p = 0.013); PALS (r:0.605; p = 0.001), and PACS (r:0.604; p = 0.001). There was a negative correlation between postcontrast posterior T1 relaxation durations and LAVI (r: − 0.467; p = 0.016). Also, there were positive correlations between postcontrast posterosuperior T1 relaxation durations and LVEF (r:0.487; p = 0.012); PACS (r:0.401; p = 0.043). The detailed results of correlation analyze between atrial T1 relaxation durations and echocardiographic findings were presented in Table 4.

### 3.6. Correlations between biomarkers and transthoracic echocardiographic findings

The negative correlation between central FGF-23 and LVEF (r: − 0.450; p = 0.013); and the positive correlation between central FGF-23 and LAVI (r:0.338; p = 0.034) were observed. Other correlation analyzes between biomarkers and echocardiographic findings were listed in Table 5.

### 3.7. Changes in transthoracic echocardiographic parameters during follow-up

In patients who had no recurrences during follow-up, left atrial mechanics were reevaluated at a 12-months follow-up visit. There was no difference among several echocardiographic parameters, which are LVEF, LV-GLS, LAVI, LAEmF, LAAEmF, LAPEmF, mean PALS, and mean PACS during follow-up visits compared to baseline parameters. There is only a significant decrease in CSI at the 12th-month visit compared to preprocedural measurement (56.7 (32–76) vs. 47.5 (31–65); p = 0.003). The details of comparative echocardiography values were shown in supplementary Table 2.

## 4. Discussion

The significant findings of our study were as follows: there were negative correlations between atrial T1 relaxation durations and FGF-23 levels and positive correlations between atrial T1 relaxation durations and PACS values.

C-MRI is an emerging tool to evaluate the underlying atrial tissue in patients with AF. In most C-MRI studies, late gadolinium enhancement was used to predict the presence of atrial fibrosis. The thin nature of atrial myocardial tissue as well as the difficulties relevant to the quantitative measurement of scar regions are essential limitations of the DE-MRI studies [16]. On the other hand, T1 mapping is a novel method to estimate atrial fibrosis. This method is mainly based on the measurement of pre-contrast and post-contrast T1 relaxation durations during the C-MRI. It is known that T1 relaxation duration is getting shorter after contrast injection [17]. Several studies demonstrated that myocardial fibrosis may cause a shorter T1 relaxation duration than normal myocardium [11]. In a previous study, Liang-Han Ling et al. showed that T1 relaxation duration is shorter in patients who have paroxysmal AF than persistent AF [9]. Moreover, they reported that the T1 relaxation duration was shorter in patients who had AF recurrence after catheter ablation. Insight of these studies, we used C-MRI T1 relaxation duration as an atrial fibrosis indicator in atrial fibrillation patients.

The thickness of atrial tissue is a significant limitation for the measurement of T1 relaxation duration, like DE-MRI. Therefore, using the thickest parts of atrial tissue, such as an interatrial septum or posterior atrium, was recommended for the measurement of T1 relaxation duration [8]. Liang-Han Ling et al. and Roy Beinart et al. used the posterior atrium and interatrial septum in their studies [9, 11]. We used the posterior and posterosuperior atrium and aimed to examine the value of different locations for atrial fibrosis prediction.

Several studies investigated the implications of biomarkers such as TGF-β1, ferritin, PTH, CA – 125, micro RNAs, N-terminal pro-B type natriuretic peptide (NT-proBNP), and b type natriuretic peptide (BNP) as a surrogate marker of fibrosis in AF patients [18–22]. Also, FGF-21 and FGF-23, members of the fibroblast growth factor family, were evaluated in previous studies for their levels in patients with AF[23, 24]. FGF-21 is an endocrine, metabolic regulator, and controls glucose and lipid homeostasis. Some trials reported that systemic and locally produced FGF-21 has protective roles on the heart, especially in hypertensive patients [25, 26]. In addition, FGF-21 levels were found to be associated with systolic dysfunction, and its expression was reported to be increased in response to the inflammation [27, 28]. Several population-based studies and meta-analyses reported that FGF-21 is associated with AF [13, 20, 23].

FGF-23 is a paracrine factor that is mainly secreted by osteocytes [29]. Some trials reported that osteoblasts, hypothalamus, thalamus, and heart could secrete FGF-23[29, 30]. We know that FGF-23 is a promoting factor for cardiac fibrosis, but its value for the prediction of recurrence after catheter ablation is still controversial[30, 31]. Begg GA. et al. reported that serum FGF-23 levels had no significant predictive value for recurrence after catheter ablation in paroxysmal, persistent, or long-standing-persistent AF patients [32]. On the other hand, they also reported serum FGF-23 levels might be associated with recurrence after cardioversion of AF, in another study [33]. We used the serum, which was derived from the femoral artery and left atrium, FGF-21, and FGF-23 as a biomarker of atrial fibrosis because of previous controversial findings. According to the previous studies, C-MRI postcontrast T1 relaxation durations are an important indicator for cardiac fibrosis. Our findings showed that especially central FGF-23 levels were in negative correlation with atrial postcontrast T1 relaxation duration, and this finding supports the significance of atrial postcontrast T1 relaxation duration and central FGF-23 levels.

In a previous study, Schaaf et al. [34] reported that LAVI is higher, and both LAAEmF and LAPEmF are lower in patients who have paroxysmal AF than healthy subjects. In another study by Im et al.[35], LAVI_max_, LAVI_min_, LAEF, LAVI_max_/LAEF, and LAVI_min_/LAEF predicted ATa recurrence after catheter ablation of AF. Although measurement of left atrial strain is a promising method for the evaluation of atrial mechanics, operator-dependence and resolution of imaging seem to be major limitations of this method [36]. Previous studies reported that patients who have peak atrial strain values higher than 19.5%–23% had lower ATa recurrence risk after catheter ablation of AF, using PALS measurement [37, 38]. In another study, Parwani et al. reported that patients who had PALS values lower than 10% are under higher ATa recurrence risk. These studies demonstrated the great variability in the measurements of PALS to predict ATa recurrence [39]. In our study, we found that LVEF, PALS, and PACS are in correlate positively with atrial post-contrast T1 relaxation durations. On the other hand, there were no correlations between atrial T1 relaxation durations and left atrial volume parameters, such as LAEmF, LAPEmF, and LAEF. We think the main reason for this finding is the volume parameters are affected later than the atrial fibrosis process. Also, the reason for the correlations between atrial strain parameters and atrial T1 relaxation durations could be a direct evaluation of atrial fibrosis with atrial strain imaging.

In our study, we observed that there was a statistically significant positive correlation between C-MRI T1 relaxation time and LVEF, LAPEmF, PALS, PACS. Likewise, there were negative correlations between C-MRI T1 relaxation time, and LAVI and CSI. We thought that this finding could be important. Because we know that we examined the single part of the atrium with C-MRI, such as a posterior or posterosuperior atrium. On the other hand, other left atrial echocardiographic findings were related to global atrial function. Correlation analyses of biomarkers and C-MRI T1 relaxation duration were similar. There were negative correlations between C-MRI T1 values and central and peripheral FGF-23 levels.

Our study has several strengths mainly related to the methods to evaluate atrial fibrosis. First of all, our study was designed as a prospective cohort study. There were previous studies that examined the atrial tissue and atrial remodeling with different techniques. However, our research is the first prospective designed study that aimed to investigate the correlation of three different methods, C-MRI, atrial strain imaging, and biomarker in the same patients. Despite the small size of the cohort, we observed similar results with prior studies. Another important strength of our study is that we have the measurement of atrial FGF 21 and 23. Most of the studies that evaluated the association between cardiac disorders and levels of these biomarkers did focus only on systemic levels. In our study, we measured both atrial and systemic levels and found that correlations between central FGF 23 levels and postcontrast T1 relaxation durations were more significant than others. We believe this finding adds another originality to our paper.

We had several limitations regarding our study. Firstly, our study population was small. We observed recurrence in only 4 of 30 patients, which created a disproportionate number of patients between the groups. Secondly, our study does not contain healthy subjects, and information about the atrial fibrosis indicators, that we used, in a healthy population is limited. New trials are needed to estimate the difference in these indicators between patients with paroxysmal atrial fibrillation and healthy subjects. Thirdly, it was hard to examine all the study population at the same time with different methods. In four patients, C-MRI could not be performed due to reasons such as claustrophobia, obesity, and acute paroxysmal atrial fibrillation episode. Although valuable information can be gathered from C-MRI analysis, the cost of the MRI, operator-dependence for the measurements, and lack of standardized methods are major concerns.

## 5. Conclusion

Atrial fibrosis could be evaluated with different techniques and the significance of these was shown in previous studies. Our studies showed that there are correlations between T1 mapping of atrial tissue, biomarkers, and atrial mechanics evaluated with transthoracic echocardiography. Our study and the correlations we found to support the significance of three different techniques aimed to investigate atrial fibrosis.

**Supplementary Table 1:** Intra-observer Reliability

Variable	ICC	95% Limit of Agreement
PALS	0.953	0.907 to 0.977
PACS	0.965	0.928 to 0.983
Variable	LCC	95% Limit of Agreement
Postcontrast T1 Relaxation Durations of Posterior Atrium	0.953	0.893 to 0.977
Postcontrast T1 Relaxation Durations of Posterosuperior Atrium	0.957	0.905 to 0.979

PALS: Peak Atrial Longitudinal Strain, PACS: Peak Atrial Contraction Strain, ICC: Intraclass Correlation Coefficient, LCC: Lin’s Correlation Coefficient

**Supplementary Table 2:** Changes in Transthoracic Echocardiographic Parameters During Follow-up

Transthoracic Echocardiography Values (n=26)		Preprocedural Total (n=30)	Preprocedural (n=26)	Postprocedural (n=26)	p
LVEF (%)	Median (Min-Max)	61 (29–73)	61.5 (47–73)	60 (48–77)	0.119
LV-GLS (%)	Median (Min-Max)	−19.7 (−8.6 / −26.7)	−19.9 (−13 / −26.7)	−19 (−14 / −27)	0.804
LAVI (mL/m2)	Median (Min-Max)	26.5 (15–70)	23 (15–64)	22 (13–55)	0.200
LAEmF (%)	Median (Min-Max)	36.5 (22–65)	37.5 (23–65)	38 (15–58)	0.614
LAAEmF (%)	Median (Min-Max)	27.5 (8–62)	28 (8–62)	29 (8–48)	0.345
LAPEmF (%)	Median (Min-Max)	11 (3–40)	10 (3–40)	15 (4–39)	0.223
Mean PALS (%)	Median (Min-Max)	20.2 (6.7–43.3)	21.3 (6.7–43.3)	21.3 (8.7–37.7)	0.917
Mean PACS (%)	Median (Min-Max)	12 (2.3–18.3)	12 (4–18.3)	10.8 (5.7–16.7)	0.054
CSI (%)	Median (Min-Max)	58 (32–78)	56.7 (32–76)	47.5 (31–65)	0.003 **

Paired Samples t Test

** p<0.01

LVEF: Left Ventricle Ejection Fraction; LV-GLS: Left Ventricle Global Longitudinal Strain; LAVI: Left atrial volume index; LAEmF: Left atrial emptying fraction; LAPEmF: Left atrial passive emptying fraction; LAAEmF: Left atrial active emptying fraction; PALS: Peak atrial longitudinal strain; PACS: Peak atrial contraction strain; CSI: Atrial contraction strain index

## Figures and Tables

**Table 1:** Baseline characteristics

Variable		(n = 30)
Sex (n, %)	Female	17, 56.7%
Age (Years)	Mean ± SD	55.23 ± 12.37
BMI (kg/m^2^)	Mean ± SD	27.92 ± 4.54
Smoking	Present	8 (26.6)
CHA2DS2-VASc	Mean ± SD	2.2 ± 1.32
Comorbidities	Hyperlipidemia	19 (63.3)
	Coronary Heart Disease	18 (60.0)
	Hypertension	17 (56.7)
	History of Cerebrovascular Event	2 (6.7)
	Diabetes Mellitus	2 (6.7)
	COPD / Asthma	2 (6.7)
	Heart Failure	1 (3.3)
Oral Anticoagulant Use	Present	19 (63.3)
Medications	Statins	15 (50.0)
	RAAS Blockers	14 (46.6)
	Metformin	2 (6.6)
	Insulin	0 (0.0)
	Beta-blockers	26 (86.6)
	Dihydropyridine Ca Channel Blockers	4 (13.3)
Blood Analysis	Hemoglobin (gr/dL)	14.1 ± 1.8
	White blood cell (n x 103)	7.2 ± 1.94
	Glomerular Filtration Rate (%)	83.56 ± 11.3
	CRP (mg/L)	0.37 ± 0.3
	BNP (pg/mL)	37.5 ± 33.5

BMI: Body Mass Index; CHA2DS2-VASc: Congestive Heart Failure, Hypertension, 2 points for Age ≥ 75 years, Diabetes Mellitus, 2 points for Stroke, Vascular Disease, 1 point for Age 75 years> ≥65 years, Gender; COPD: Chronic Obstructive Pulmonary Disease; RAAS: Renin Angiotensin Aldosterone System; CRP: C-Reactive Peptide; BNP: Brain Natriuretic Peptide.

**Table 2:** Results of the techniques selected to evaluation of atrial fibrosis.

Cardiac magnetic resonance imaging (n = 26)			
	Precontrast T1 Relaxation Durations (ms), Median (Min-Max)	Postcontrast T1 Relaxation Durations (ms), Median (Min-Max)	
Posterior	1004 (614–1749)	428 (292–540)	
Posterosuperior	903.5 (462–1456)	373 (288–718)	
Atrial Strain Values (n=30)			
PALS (%), Median (Min-Max)	20.2 (6.7–43.3)	PACS (%), Median (Min-Max)	12 (2.3 – 18.3)
Biomarkers (n=30)			
	FGF-21 (pg/mL), Median (Min-Max)	FGF-23 (pg/ml), Median (Min-Max)	
Peripheral	223.5 (34–762)	1981.8 (1166.7–2604.2)	
Central	187 (37–531)	1865 (1208–2541.7)	

PALS: Peak Atrial Longitudinal Strain; PACS: Peak Atrial Contraction Strain; FGF-21: Fibroblast Growth Factor – 21; FGF-23: Fibroblast Growth Factor - 23.

**Table 3:** Correlation Between Biomarkers and Atrial T1 Relaxation Durations

N = 26	Peripheral FGF-23 (pg/mL)		Central FGF-23 (pg/mL)	Peripheral FGF-21 (pg/mL)	Central FGF-21 (pg/mL)
Precontrast MRI T1 Relaxation Duration (Posterior)	r	−0.384	−0.229	−0.091	−0.046
	*p*	*0.053*	*0.261*	*0.657*	*0.825*
Postcontrast MRI T1 Relaxation Duration (Posterior)	r	−0.451	−0.561	0.045	0.018
	*p*	*0.021* *	*0.003* **	*0.827*	*0.929*
Precontrast MRI T1 Relaxation Duration (Posterosuperior)	r	−0.311	-0.322	0.374	0.429
	*p*	*0.122*	*0.108*	*0.060*	*0.029* *
Postcontrast MRI T1 Relaxation Duration (Posterosuperior)	r	−0.250	−0.624	0.293	0.283
	*p*	*0.218*	*0.001* **	*0.147*	*0.162*

r: Pearson Correlation Coefficient

* p < 0.05

** p < 0.01

**Table 4:** Correlation between cardiac MRI T1 relaxation durations and echocardiographic values.

		Precontrast MRI T1 Relaxation Duration (Posterior)	Postcontrast MRI T1 Relaxation Duration (Posterior)	Precontrast MRI T1 Relaxation Duration (Posterosuperior)	Postcontrast MRI T1 Relaxation Duration (Posterosuperior)
*Preprocedural (n=26)*					
LVEF (%)	r	0.048	0.671	0.300	0.487
	*p*	*0.816*	*0.001* **	*0.137*	*0.012* *
LV-GLS (%)	r	0.157	0.343	0.430	0.321
	*p*	*0.445*	*0.086*	*0.028* *	*0.110*
LAVI (mL/m^2^)	r	−0.307	−0.467	−0.070	−0.075
	*p*	*0.127*	*0.016* *	*0.735*	*0.715*
LAEmF (%)	r	0.134	0.171	−0.152	−0.056
	*p*	*0.514*	*0.404*	*0.460*	*0.786*
LAAEmF (%)	r	0.219	0.482	−0.129	0.149
	*p*	*0.283*	*0.013* *	*0.530*	*0.467*
LAPEmF (%)	r	−0.186	−0.215	−0.356	−0.177
	*p*	*0.362*	*0.292*	*0.074*	*0.387*
Mean PALS (%)	r	0.032	0.605	0.065	0.340
	*p*	*0.878*	*0.001* **	*0.752*	*0.089*
Mean PACS (%)	r	0.045	0.604	0.152	0.401
	*p*	*0.828*	*0.001* **	*0.459*	*0.043* *
CSI (%)	r	−0.113	−0.497	0.029	−0.115
	*p*	*0.581*	*0.010* *	*0.888*	*0.575*

r: Pearson Correlation Coefficient

* p < 0.05

** p < 0.01

LVEF: Left Ventricle Ejection Fraction; LV-GLS: Left Ventricle Global Longitudinal Strain; LAVI: Left atrial volume index; LAEmF: Left atrial emptying fraction; LAPEmF: Left atrial passive emptying fraction; LAAEmF: Left atrial active emptying fraction; PALS: Peak atrial longitudinal strain; PACS: Peak atrial contraction strain; CSI: Atrial contraction strain index.

**Table 5:** Correlation between biomarkers and echocardiographic values.

		Peripheral FGF-23 (pg/mL)	Central FGF-23 (pg/mL)	Peripheral FGF-21 (pg/mL)	Central FGF-21 (pg/mL)
*Preprocedural (n = 30)*					
LVEF (%)	r	−0.473	−0.450	0.058	0.101
	*p*	*0.008* **	*0.013* *	*0.759*	*0.594*
LV-GLS (%)	r	−0.246	−0.249	0.103	0.151
	*p*	*0.189*	*0.184*	*0.589*	*0.424*
LAVI (mL/m^2^)	r	0.452	0.388	0.335	0.140
	*p*	*0.012* *	*0.034* *	*0.071*	*0.460*
LAEmF (%)	r	−0.174	−0.154	−0.188	−0.120
	*p*	*0.358*	*0.417*	*0.319*	*0.528*
LAAEmF (%)	r	−0.421	−0.359	−0.169	−0.129
	*p*	*0.020* *	*0.051*	*0.372*	*0.495*
LAPEmF (%)	r	0.360	0.336	−0.142	−0.157
	*p*	*0.051*	*0.069*	*0.454*	*0.406*
Mean PALS (%)	r	0.070	−0.100	−0.179	−0.137
	*p*	*0.714*	*0.598*	*0.343*	*0.470*
Mean PACS (%)	r	0.022	−0.207	0.070	0.072
	*p*	*0.906*	*0.273*	*0.715*	*0.706*
CSI (%)	r	0.209	0.162	0.294	0.183
	*p*	*0.268*	*0.391*	*0.115*	*0.332*

r: Pearson Correlation Coefficient

* p<0.05

** p<0.01

LVEF: Left Ventricle Ejection Fraction; LV-GLS: Left Ventricle Global Longitudinal Strain; LAVI: Left atrial volume index; LAEmF: Left atrial emptying fraction; LAPEmF: Left atrial passive emptying fraction; LAAEmF: Left atrial active emptying fraction; PALS: Peak atrial longitudinal strain; PACS: Peak atrial contraction strain; CSI: Atrial contraction strain index
